# Supplementary material for: A fault-intrusion-tolerant system and deadline-aware algorithm for scheduling scientific workflow in the cloud
Source: PeerJ Comput Sci. 2021 Nov 2;7:e747. doi: 10.7717/peerj-cs.747 (PMC8576554; doi:10.7717/peerj-cs.747)
Supplement: Supplemental Information 6 [file peerj-cs-07-747-s006.zip › Myproject1.1/docs/org/cloudbus/cloudsim/distributions/GammaDistr.html]

GammaDistr


JavaScript is disabled on your browser.


- Overview
- Package
- Class
- Use
- Tree
- Deprecated
- Index
- Help

- Prev Class
- Next Class

- Frames
- No Frames

- All Classes

- Summary:
- Nested |
- Field |
- Constr |
- Method

- Detail:
- Field |
- Constr |
- Method


org.cloudbus.cloudsim.distributions

## Class GammaDistr

- java.lang.Object
- - org.cloudbus.cloudsim.distributions.GammaDistr

- All Implemented Interfaces:
  :   ContinuousDistribution

  ---

    

  ```
  public class GammaDistr
  extends java.lang.Object
  implements ContinuousDistribution
  ```

  The Class GammaDistr.

  Since:
  :   CloudSim Toolkit 1.0

- - ### Constructor Summary

    Constructors

    Constructor and Description

    `GammaDistr(int alpha,
    double beta)`

    Instantiates a new gamma distr.

    `GammaDistr(java.util.Random seed,
    int alpha,
    double beta)`

    Instantiates a new gamma distr.
  - ### Method Summary

    Methods

    | Modifier and Type | Method and Description |
    | --- | --- |
    | `double` | `sample()` Sample the random number generator. |

    - ### Methods inherited from class java.lang.Object

      `clone, equals, finalize, getClass, hashCode, notify, notifyAll, toString, wait, wait, wait`

- - ### Constructor Detail


    - #### GammaDistr

      ```
      public GammaDistr(java.util.Random seed,
                int alpha,
                double beta)
      ```

      Instantiates a new gamma distr.

      Parameters:
      :   `seed` - the seed
      :   `alpha` - the alpha
      :   `beta` - the beta


    - #### GammaDistr

      ```
      public GammaDistr(int alpha,
                double beta)
      ```

      Instantiates a new gamma distr.

      Parameters:
      :   `alpha` - the alpha
      :   `beta` - the beta
  - ### Method Detail


    - #### sample

      ```
      public double sample()
      ```

      **Description copied from interface: `ContinuousDistribution`**

      Sample the random number generator.

      **Specified by:**
      :   `sample` in interface `ContinuousDistribution`

      Returns:
      :   The sample


- Overview
- Package
- Class
- Use
- Tree
- Deprecated
- Index
- Help

- Prev Class
- Next Class

- Frames
- No Frames

- All Classes

- Summary:
- Nested |
- Field |
- Constr |
- Method

- Detail:
- Field |
- Constr |
- Method
